# Tracheal collapse diagnosed by multidetector computed tomography: evaluation of different image analysis methods

**DOI:** 10.1080/20018525.2017.1407624

**Published:** 2017-12-08

**Authors:** Mette Nygaard, Elisabeth Bendstrup, Ronald Dahl, Ole Hilberg, Finn Rasmussen

**Affiliations:** a Department of Respiratory Diseases and Allergy, Aarhus University Hospital, Aarhus, Denmark; b Department of Radiology, Aarhus University Hospital, Aarhus, Denmark; c Department of Respiratory Medicine, Odense University Hospital, Odense C, Denmark

**Keywords:** excessive tracheal collapse, diagnosis, MDCT, prevalence, correlation, pulmonary function test

## Abstract

**Background**: The gold standard for diagnosing excessive tracheal collapse is still evaluation during bronchoscopy. Today, multidetector computed tomography (MDCT) is used to confirm a suspicion of abnormal tracheal collapse. There is no gold standard for computed tomography (CT) image analysis of tracheal collapse.

**Purpose**: To evaluate four different methods for the diagnosis of tracheal collapse using the images obtained through MDCT to help clinicians evaluate the images in daily practice.

**Objectives**: 374 consecutive high-resolution CT scans with full inspiratory and end-expiratory CT scans were retrospectively analyzed.

**Methods**: The images were analyzed in four different ways. The degree of collapse was based on cross-sectional areas of individual locations or volumes of entire regions: (1) 1 cm above the carina, (2) the level of maximal collapse of the trachea, (3) the entire region from the carina to the thoracic inlet, and (4) the trachea and bronchial region as defined by the software.

**Results**: We compared three existing and one new method for image analysis of tracheal collapse by MDCT. The prevalence of tracheal collapse varied from 10.7% to 19.5% in this cohort of patients suffering from mixed lung diseases when using an expiratory collapse of ≥50% as a threshold. The four methods were comparable with highly significant Pearsons correlation coefficients (0.764–0.856). However, the four methods identified different patients with collapse of ≥50%. There was no correlation between symptoms and the degree of collapse.

**Conclusion**: The different methods identify tracheal collapse in different patients. Hence, the diagnosis of excessive tracheal collapse can not rely solely on MDCT images. Generally, there is a poor correlation between symptoms and the degree of collapse in the different methods. However, when using the maximal collapse, there is some correlation with symptoms. When in doubt regarding the diagnosis, further investigations, such as bronchoscopy, should be carried out.

## Introduction

Excessive tracheal collapse is characterized by flaccidity of the tracheal wall resulting in expiratory collapse of ≥50% of the central airways. The most frequent symptoms of tracheal collapse are cough, dyspnea, wheezing, and recurrent infections. This complicates diagnosis, as it mimics other and more common lung diseases [1].

**Figure 1.**
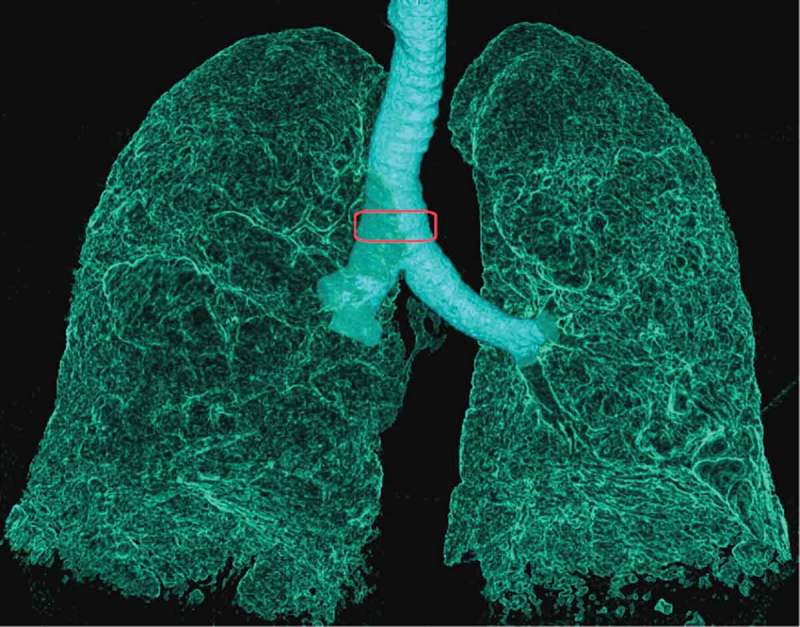
Inspiratory sagittal reconstructed CT images of the air in the lungs and airways explaining Method 1: measurement of the cross-sectional area 1 cm above the carina.

**Figure 2.**
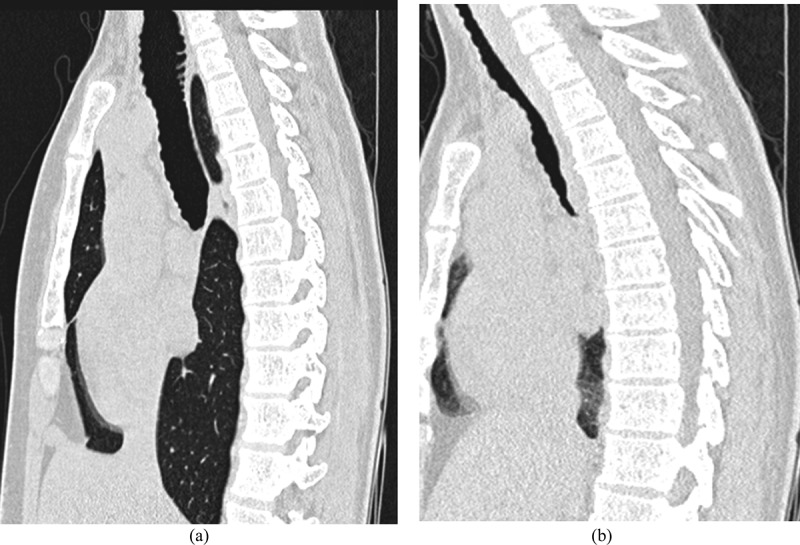
Method 2: paired end-inspiratory (a) and end-expiratory (b) sagittal reconstructed CT images showing maximal collapse of more than 50%.

**Figure 3.**
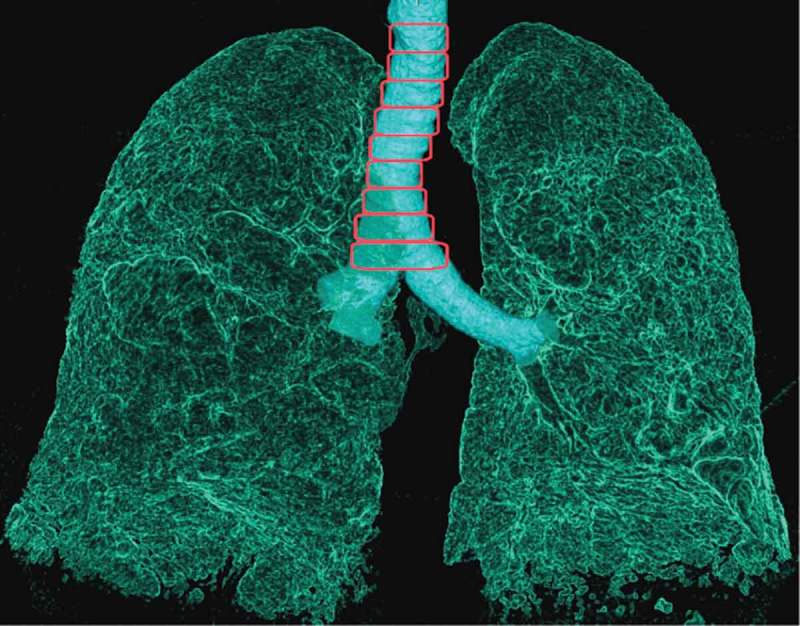
Inspiratory sagittal reconstructed CT images of the air in the lungs and airways explaining Method 3: measurement of the cross-sectional area every centimeter from the carina to the thoracic outlet.

**Figure 4.**
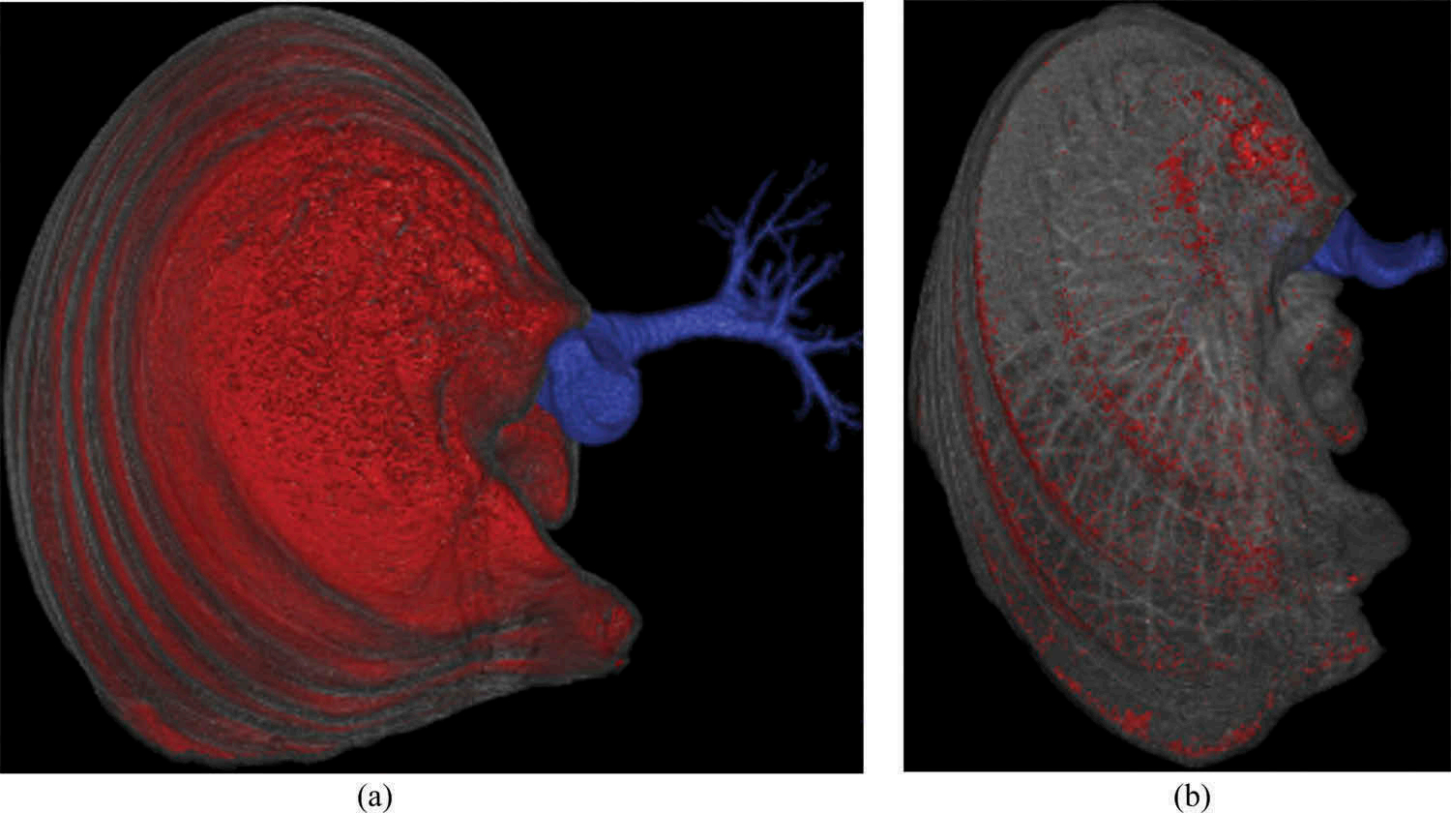
Automatic segmentation of the lungs and the airways at (a) full inspiration and at (b) end expiration. The red area shows the areas of the right lung with HU above −950 (inspiration) and −800 (expiration) respectively. The volume of the trachea and first generations of bronchi given by the software (Method 4) is shown in blue.

**Figure 5.**
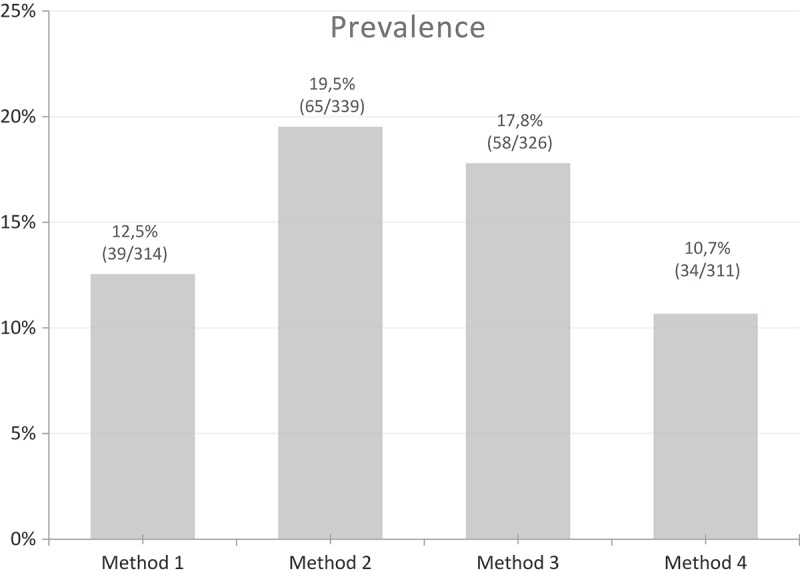
Prevalence of tracheomalacia using the four different methods and a threshold of 50% for maximal airway collapse.

**Figure 6.**
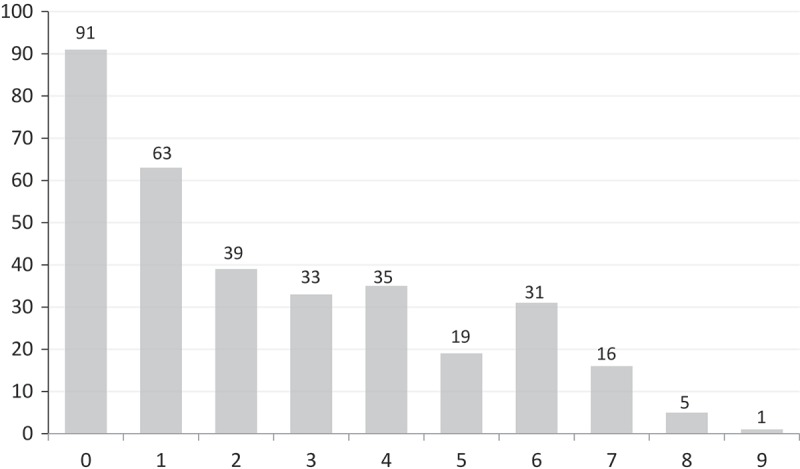
Histogram of the number of patients with maximal collapse of the tracheal cross-sectional area at the carina (0) and every 1 cm until the thoracic inlet. Numbers are based on measurements from Method 3.

**Figure 7.**
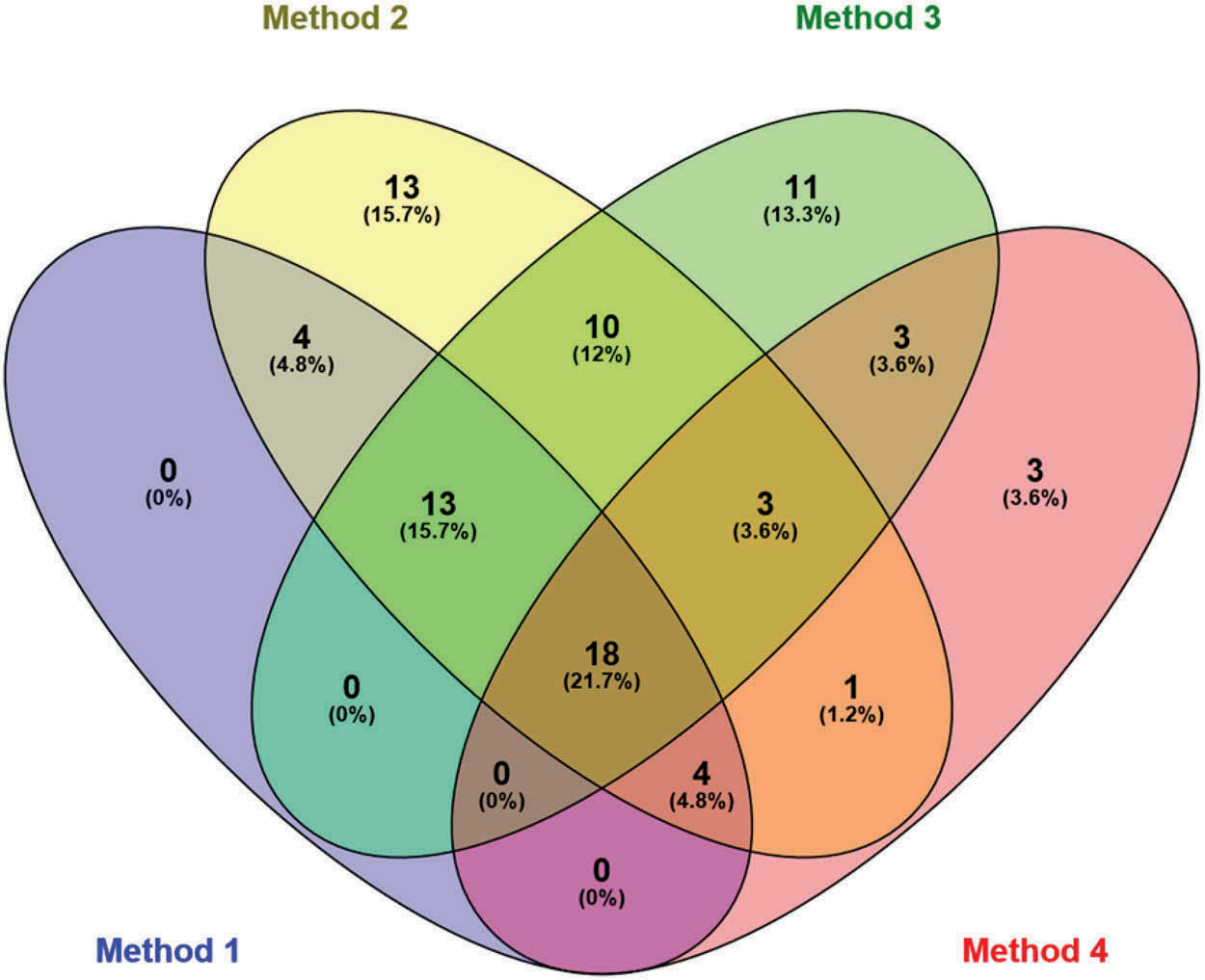
Venn diagram showing the overlap between the patients with collapse of more than 50% in expiration using the four different methods.

The reported prevalence of tracheal collapse varies in different studies and depends both on the method used for diagnosing tracheomalacia and on the comorbidities of the patients [2–5]. The highest prevalence reported is 23% in patients with chronic bronchitis. In this single study, the diagnosis was solely based on bronchoscopy [2]. Another study reported a prevalence of 14.1% in patients with chronic cough [3].

The gold standard for diagnosing excessive tracheal collapse is still evaluation of the cross-sectional area of the trachea before and at forced expiration or cough during bronchoscopy. Until recently, this was the only available diagnostic option. With the introduction of multidetector computed tomography (MDCT), a non-invasive diagnostic tool is now available. This new tool poses new challenges such as standardization of a scanning protocol, standardization of image analysis, definition of the degree of airways collapse, and determination of the threshold for normal/abnormal airway collapse. Several methods for tracheal collapse evaluation of MDCT images have previously been used [6–8]. The most commonly used method is the determination of airway collapse at the localization of maximal collapse of the trachea [3,7,9]. In other methods, predetermined locations are used for establishing the degree of collapse in the trachea [4,5]. These locations differ, but most include levels 1 cm above the carina and the aortic arch [6–10].

There is no gold standard for computed tomography (CT) images analysis of tracheal collapse. To our knowledge, no studies have previously compared different image analysis methods with respect to identification of tracheal collapse. The aim of the present study was therefore to compare four different methods of high-resolution CT image analysis for tracheal collapse to help the clinician to appraise the CT images in the daily practice.

## Materials and methods

### Patients

This study was approved by the Danish Data Protection Agency. During a 17-month period, 374 patients were referred to a high-resolution computed tomography (HRCT) scan performed in full inspiration and in end expiration. No participants were recruited exclusively for this study. The volume of tracheal collapse was measured retrospectively. The indication for HRCT is shown in Table 1.

**Table 1.** Indication for performing the HRCT scans.

Indication	Number	Percentage
ILD^a^	175	49.6
Emphysema (COPD)	14	4
Bronchiectasis	80	22.6
Tracheomalacia	16	4.5
Infiltrates	7	2.0
LTX, BOS	2	0.6
Dyspnea	9	2.6
Other	23	6.5
Combination of two of the above	16	4.5

ILD = interstitial lung disease; COPD = chronic obstructive lung disease; LTX = lung transplant; BOS = bronchiolitis obliterans syndrome.

^a^Known or referred on the suspicion of ILD.

Patient files were scrutinized for data on age, respiratory symptoms (dyspnea, cough, and recurrent infection), smoking history, body mass index and pulmonary function data [forced expiratory volume in 1 s (FEV1), forced vital capacity (FVC), FEV1/FVC, forced expiratory flow (FEF25–75)] at 3 months before and 3 months after the HRCT. Pulmonary function tests were analyzed in accordance with American Thoracic Society and European Respiratory Society guidelines [11] (Table 2).

**Table 2.** Descriptive characteristics of the study population by the four different methods.

	Method 1		Method 2		Method 3		Method 4	
	Collapse ≥50	Collapse ‹50	Collapse ≥50	Collapse ‹50	Collapse ≥50	Collapse ‹50	Collapse ≥50	Collapse ‹50
Age, years	64*	56*	64*	56*	64*	56*	63*	56*
Gender:								
Female, *n* (%)	29* (9)	150* (49)	43 (13)	144 (44)	41* (12)	146* (45)	23 (8)	145 (48)
Male, *n* (%)	9* (3)	125* (39)	21 (6)	124 (37)	17* (5)	122* (37)	9 (3)	123 (41)
BMI	26.6	26.2	27.7*	25.8*	27.6*	25.9*	26	26
Lung function parameters:								
FEV1 (%predicted)	75	78	73	78	79	77	79	78
FVC (%predicted)	87	88	84	89	90	87	90	88
FEV1/FVC	71	72	70	72	71	72	72	71
Flow-volume-loop								
with oscillation, n(%)	9* (67)	25* (16)	11 (30)	26 (16)	9 (26)	27 (17)	8*(38)	25*(16)
Symptoms:								
Dyspnea, *n* (%)	26 (87)	164 (72)	44* (85)	158* (71)	39 (83)	159 (72)	23 (77)	159 (72)
Cough, *n* (%)	21 (72)	138 (67)	34 (72)	134 (66)	31 (69)	131 (66)	17 (63)	135 (66)
Rec.inf., *n* (5)	13 (45)	69 (35)	22 (46)	62 (32)	16 (37)	65 (34)	10 (38)	66 (34)
Tobacco:								
Current, *n* (%)	3 (16)	30 (16)	6 (17)	34 (18)	2 (7)	36 (19)	3 (18)	37 (19)
Former, *n* (%)	9 (47)	85 (45)	18 (51)	83 (44)	17 (55)	80 (42)	7 (42)	84 (44)
Never, *n* (%)	7 (37)	74 (39)	11 (31)	73 (38)	12 (39)	71 (38)	7 (42)	72 (37)

Method: 1: 1 cm above the carina; 2: max collapse; 3: sum of cross-sectional areas; 4: volume. FEV1 = forced expiratory volume in 1 s; FVC = forced vital capacity; FEF = forced expiratory flow.

*p < 0.05.

### CT scan

HRCT was performed on a multirow detector scanner (Philips Brilliance CT 64 or ICT 256, Philips Healthcare, Best, The Netherlands) and included a scan range of the whole chest in full inspiration and at end expiration. The CT acquisition parameters were 64 × 0.625 mm or 126 × 0.625 mm collimation, kV 120, mAs/slice 150–200, rotation time 0.5 s, pitch 0.59, reconstruction thickness 0.9 mm, and increment 0.45 mm. A high-resolution kernel was used. Images were transferred to a picture-archiving communication system.

During the scanning acquisition, an automatic voice recording instructed the patients to take a deep breath and hold it for end-inspiratory scanning. For end-expiratory scanning, the patients were instructed to take a deep breath, exhale, and hold it.

### Image interpretation

Image assessment was performed on a dedicated CT workstation (Philips, Extended Brilliance workspace version 4.5.2) using standard lung window display settings (level: –500  hounsfield units (HU); width: 1500 HU). The images were retrospectively analyzed by a registrar in pulmonary medicine (M.N., with 5 years of experience in interpreting dynamic thoracic CT scans). Four different methods for calculation of tracheal collapse were performed:

### Method 1

The volume of a 10 mm CT slice located 1 cm above the carina as shown in Figure 1.

### Method 2

The volume of 10 mm CT slices from the level of the carina and every 1 cm until the thoracic inlet was examined. The location with maximal collapse was chosen as depicted in Figure 2.

### Method 3

Each 10 mm slice from the carina to the thoracic outlet was manually defined on both inspiratory and expiratory scans. The sum of these volumes was multiplied (Figure 3).

### Method 4

An automatic software segmentation technique of the lungs and the large airways was used to estimate the volume of the trachea (lung density, brilliance extended workspace) in the inspiratory and expiratory scan (Figure 4). The upper limit for the segmentation was the thoracic inlet. The lower limit was the carina. This method is fully automated and therefore without interobserver variability.

The relative collapse in expiration was calculated as:

[(luminal area or volume at inspiration—luminal area or volume at expiration)/luminal area or volume at inspiration] × 100%

### Statistical analysis

Normal distribution of all variables was confirmed by Q–Q plots. The correlation between the different methods was primarily evaluated using Bland–Altman plots. Afterwards, the Pearson correlation coefficient was used to evaluate the correlation between the different strategies for analyzing the images.

Sample characteristics were examined, and continuous variables were compared by the unpaired *t*-test. The tests are considered one-sided, as an excessive tracheal collapse never would lead to an improvement in the pulmonary function tests. The categorical variables were analyzed using the chi-square analysis.

The level of significance was set at *p* < 0.05 for all statistical analyses. All analyses were performed using statistical software (STATA, version 13).

## Results

### Study population

A total of 353 HRCT scans met the inclusion criteria. The cohort consisted of 191 females (median age 55 years, range 14–91 years) and 150 males (median age 60 years, range 18–88 years). Patient demographics are listed in Table 2. Information on symptoms and spirometry was available in 225 subjects.

### Prevalence

The prevalence of tracheal collapse varied from 10.7% to 19.5% when using an expiratory collapse of 50% as a threshold (Figure 5). The prevalences were significantly different from each other (*p* < 0.001). Using an expiratory collapse of 80% as threshold, only 1–3 patients were identified, and the prevalence decreased to <1% for all four methods.

There was no significant difference between the prevalence of abnormal collapse in the three major lung disease groups: asthma (4.55–19.57), chronic obstructive pulmonary disease (COPD) (10.42–28.30), and interstitial lung diseases (13.33–19.75) (*p* = 0.289–0.817, chi-square analysis) using three out of four methods.

### Measurement of the tracheal collapse

The cross-sectional area was calculated every 1 cm from the carina to the thoracic inlet (Method 3) to determine the localization of maximal expiratory collapse (Method 2). The maximal collapse was located at the carina in 27% of patients (Figure 6). When evaluating the area at the carina and the lower 4 cm of the trachea, maximal collapse was identified in 78%. Thus, in our population, tracheal collapse was most pronounced in the distal part of the trachea.

Correlations between the four different methods for measuring tracheal collapse were analyzed using Bland–Altman plots. The plots were acceptable with a systematic difference between the methods (mean difference between 0.3 and 11 depending on compared methods). These results allowed us to use the pairwise linear regression analysis. The Pearson correlations were between *r* = 0.764 and *r* = 0.856 (*p* < 0.001).

### Correlation with age and gender

Patients with expiratory tracheal collapse of ≥50% were significantly older than patients without collapse (63 years contra 56 years), and apparent for all four measuring methods (Table 2). For both genders, the highest prevalence of abnormal tracheal collapse was found in the age group between 70 and 79 years (females 38%, males 17%). The difference in average collapse between genders was statistically significant in method 1 and method 3 with significantly more women with collapse of ≥50% (*p* = 0.014 and *p* = 0.028). In method 2 and method 4, the difference was just insignificant (*p* = 0.056).

### Correlation with symptoms, smoking, and BMI

The relationship between three common respiratory symptoms, cough, dyspnea, and recurrent respiratory infections, and tracheal collapse, was evaluated. Significantly more patients in the group with collapse of ≥50% had dyspnea using method 2 (*p* < 0.05). They also showed a trend toward recurrent infections, albeit not insignificant (*p* = 0.07). The other methods revealed no significant relation between a tracheal collapse of 50% and dyspnea, recurrent infections, or cough (Table 2). The BMI was significantly higher in the group with collapse of ≥50% (BMI: 27.6–27.8) when compared with the group without collapse (BMI: 25.9) when using methods 2 and 3 (*p* = 0.020 and 0.039, *t*-test). When using method 1 (1 cm above the carina) and method 4 (volume given by the software), there was no difference.

### Correlation with physiological measurements

There was no correlation between expiratory tracheal collapse and lung function tests using any of the four image-analysis methods (Table 3). However, we noticed a trend toward a correlation between the tracheal collapse and the FEV1 and FVC when using method 2. The FEV1 and FVC were insignificantly (*p* = 0.06 and *p* = 0.09) lower in the group with tracheal collapse compared with the patients with normal tracheal function. The patients with tracheal collapse of ≥50% had a tendency toward oscillations on the flow-volume loops. In methods 1 and 4, the number of flow-volume loops with oscillations was significantly higher in the group with abnormal collapse (≥ 50%) than in the group with less collapsibility (*p* = 0.01 and *p* = 0.005). In method 2, there was a trend (*p* = 0.06).

## Discussion

In the present study, we compared three existing and one new method for image analysis of tracheal collapse by MDCT. The four methods were comparable with highly significant correlation coefficients (0.764–0.856), but did not identify the same patients. Different groups of patients with airway collapse of ≥50% were identified depending on the method used for image analysis. Hence, in the clinical practice, it is important not to rely solely on the results of the MDCT but to investigate further, if there is a clinical suspicion of excessive tracheal collapse. There is no significant correlation between the degree of collapse seen on MDCT and clinical symptoms, making the diagnosis even harder. However, the correlation between the degree of collapse and symptoms has never been established in bronchoscopy either. In Figure 7, the overlap between the patients with tracheal collapse in the four methods is shown in a Venn diagram. It reveals no consistency between the patients diagnosed in the different methods. Thus, standardization of the MDCT method and correlation with other indices such as bronchoscopic detection of tracheal collapse and symptoms are needed.

The prevalence of tracheal collapse in our cohort of patients with different lung diseases ranged from 10.7% to 19.5% depending on the method used for image analysis. Previously, other characteristics have been included in the diagnosis such as in the multidimensional classification system Functional status, Extent, Morfology, Origin and Severity of the abnormalities (FEMOS) that includes symptoms, etiology, morphology, extent, and severity of the collapse [12].

The method resulting in the highest prevalence of abnormal collapse is naturally the one using the maximal collapse at a certain level. In our study, the area with maximal collapse is based on measurements of the cross-sectional areas from the carina to the thoracic inlet. In a study by Lee et al., a radiologist determined the maximal collapse visually, while Ochs et al. used a technique similar to ours that minimizes the interobserver variation [13,14]. An important problem with this method is that patients with only minor localized collapse are also included, and the impact of this is unknown and questionable [15]. In our study, there were no patients with previous intubation trauma. Prolonged intubation may lead to a segmental collapse of the trachea, and such a localized collapse will also be diagnosed by the method using the maximal collapse.

The method using the maximal collapse (Method 2) was the only method that revealed a significant correlation between symptoms and tracheal collapse. There was a higher percentage of patients suffering from dyspnea in the group of patients with tracheal collapse of ≥50% by the use of this method. Furthermore, there was a trend toward a higher risk of recurrent infections (*p* = 0.07), low FEV1 (*p* = 0.06), and FVC (p = 0.09) in the group with significant tracheal collapse. Methods 1 and 2 using only one location for evaluation of the collapsibility are rapid and easily performed. Previous studies have most often used the location 1 cm above the aortic arch for evaluation [7,9,10]. However, in our study, this was a rather rare location for the maximal collapse. When the method using only one level for evaluation of the collapse is applied, the most optimal location to measure is the distal levels of the trachea, as 78% of our patients had their maximal collapse there. As the clinical impact of expiratory tracheal collapse on imaging has not been determined, it is important to develop objective measurements for the diagnosis. In addition, it is important to relate bronchoscopic findings and MDCT to the symptoms presented by the patient. So far, no such studies have been published.

The method using the cross-sectional areas from the carina to the thoracic inlet is more time-consuming with more interobserver variation, but the advantage is that the whole trachea is evaluated, thus providing information about both the severity and the disease extent.

No readers of CT scans can rely only on software results. Some patients have extensive collapse making segmentation by the software impossible, and thus the radiologist has to evaluate pronounced collapse or consider other reasons for the inability for an automatic segmentation. However, when feasible, the fully automatic method is rapid without interobserver variation. Inherently, the inability of the software to generate the volume of the trachea in case of severe collapse poses major problems, as the most severely affected patients are not identified. Consequently, the correlation with symptoms and pulmonary function tests is low when using this method.

All analysis methods found a significantly higher mean age in the group with tracheal collapse above 50% compared with the group with less collapse. It is tempting to impute the weakness of the tracheal wall to the general degenerative process of aging, but this needs to be proven. The patients with tracheal collapse had a higher mean BMI. It is questionable whether a difference in mean BMI of 1.5 is of clinical importance. However, this is in accordance with a recent study reporting a significantly higher degree of collapse in patients with COPD and a BMI ≥ 35 [6]. A higher intrathoracic pressure in this group of patients is thought to play a role. Such a relationship needs to be investigated in prospective studies in which a time–volume curve of the trachea is compared with a time–pressure curve during expiration.

Previous studies have suggested 80% as the threshold for excessive tracheal collapse [10]. In our study, only a few patients were identified using an 80% threshold. The prevalence decreased to <1% independent of which of the four methods were used. The clinical significant threshold needs still to be defined, as we found no correlation between clinically relevant symptoms and the degree of collapse in the trachea.

We acknowledge that there are several limitations in our study. The study is retrospective and observational in design. The indication for the MDCT scans was not to evaluate the collapsibility of the trachea. Our study is based on MDCT scans performed to diagnose a broad variety of lung diseases. The frequency of tracheal collapse was the same in obstructive airway diseases (asthma and COPD) and interstitial lung disease. The expiratory images were obtained at end expiration instead of during forced expiration, which was previously shown to lower the prevalence [16]. We believe that our results are transferable to patients suffering from chronic lung disease in general. The prevalence of tracheal collapse in healthy humans is not known, but we considered expiratory CT in healthy volunteers to be unethical and can therefore, however unlikely, not exclude tracheal collapse to be a common radiologic finding. Speculations on whether collapse of the trachea in expiration is a stationary condition or just one of many manifestations of severe lung diseases need to be explored in longitudinal studies.

In conclusion, we have evaluated four different methods for the diagnosis of tracheal collapse using MDCT scanning. The different methods identify tracheal collapse in different patients depending on the method used. It is important to emphasize that there is a poor agreement between the four methods used for image analysis and a weak correlation between symptoms and degree of collapse. The software-generated results are easy and without interobserver variability, but do not identify severe collapse, thus making its use limited. The method using maximal collapse to identify patients reveals some correlations with both symptoms and pulmonary function tests, thus making this the method of choice for evaluating tracheal collapse on MDCT images. Research is warranted to determine which method correlates better with bronchoscopy, PFTs, or symptoms of tracheal collapse. A future study is planned to evaluate the tracheal collapse in expiration using both bronchoscopy and MDCT in different groups of patients suffering from known lung diseases. The correlation between the bronchoscopic results and the different image analysis will be examined and related to symptoms and pulmonary function tests.

It is still uncertain if tracheal collapse is an independent concurrent disease, a comorbidity, or a consequence of an underlying lung disease, and prospective studies are warranted.
